# Biosynthesis of Co_3_O_4_ nanomedicine by using *Mollugo oppositifolia* L. aqueous leaf extract and its antimicrobial, mosquito larvicidal activities

**DOI:** 10.1038/s41598-023-35877-z

**Published:** 2023-06-02

**Authors:** P. Gowthami, A. Kosiha, S. Meenakshi, G. Boopathy, A. G. Ramu, Dongjin Choi

**Affiliations:** 1 PG Department of Chemistry, Shrimati Devkunvar Nanalal Bhatt Vaishnav College for Women, Chennai, India; 2 School of Basic Sciences, Vels Institute of Science, Technology & Advanced Studies (VISTAS), Pallavaram, Chennai India; 3 Department of Chemistry, SRM Institute of Science and Technology (SRMIST), Ramapuram Campus, Chennai, 600 089 India; 4 Peri College of Arts and Science, Mannivakkam, Chennai, 600048 India; 5 Department of Materials Science and Engineering, Hongik University, 2639, Sejong-ro, Jochiwon-eup, Sejong, 30016 Republic of Korea

**Keywords:** Biochemistry, Biotechnology, Chemical biology, Microbiology

## Abstract

Nanotechnology is a relatively revolutionary area that generates day-to-day advancement. It makes a significant impact on our daily life. For example, in parasitology, catalysis and cosmetics, nanoparticles possess distinctive possessions that make it possible for them in a broad range of areas. We utilized *Mollugo oppositifolia* L. aqueous leaf extract assisted chemical reduction method to synthesize Co_3_O_4_ nanoparticles. Biosynthesized Co_3_O_4_ Nps were confirmed via UV–Vis spectroscopy, scanning electron microscope, X-ray diffraction, EDX, Fourier-transform infrared, and HR-TEM analysis. The crystallite size from XRD studies revealed around 22.7 nm. The biosynthesized Co_3_O_4_ nanoparticle was further assessed for mosquito larvicidal activity against south-urban mosquito larvae *Culex quinquefasciatus*, and antimicrobial activities. The synthesized Co_3_O_4_ particle (**2**) displayed significant larvicidal activity towards mosquito larvae *Culex quinquefasciatus* with the LD_50_ value of 34.96 µg/mL than aqueous plant extract (**1**) and control Permethrin with the LD_50_ value of 82.41 and 72.44 µg/mL. When compared to the standard antibacterial treatment, Ciprofloxacin, the Co_3_O_4_ nanoparticle (**2**) produced demonstrates significantly enhanced antibacterial action against the pathogens *E. coli* and *B. cereus*. The MIC for Co_3_O_4_ nanoparticles 2 against *C. albicans* was under 1 μg/mL, which was much lower than the MIC for the control drug, clotrimale, which was 2 µg per milliliter. Co_3_O_4_ nanoparticles 2, with a MIC of 2 μg/mL, has much higher antifungal activity than clotrimale, whose MIC is 4 μg/mL, against *M. audouinii*.

## Introduction

Chemical insecticides are used for mosquito control, but they are harmful to non-target animals and trigger human health issues. Therefore, effective and economically friendly control systems must be targeted in order to manage this challenge effectively. In order to monitor mosquitoes successfully through several mechanisms, biopesticides can be created. Globally, mosquitoes transmit terrible diseases and parasites, such as measles, dengue, filariasis, etc. Mosquitoes are common and end in almost two million lives each year^1^. Many mosquito diseases, like damage of socioeconomic and manual work in subtropical and tropical countries, consume financial power, but vector-borne diseases are not protected in the climate of earth's ecosystems^2^. *Anopheles stephensi* is the primary vector of malaria in India. Malaria, while with a previous squeak of between 1.1 and 2.7 million, has been one of the main serious infectious diseases with a prevalence estimated at 300–500 million through health manifestations. Nearly 40 percent of people living in the biosphere also remain in tropical malaria sites^3^. For between 120 and 44 million citizens worldwide, the lymphatic filariasis agent of *Culex quinquefasciatus*, usually distributed through rainfall, is a recurring phenomenon^4^.

The specific mechanism underlying the larvicidal action of AgNPs is not entirely known, considering the existence of several research articles on the topic. The smaller size of AgNPs has led some authors to believe that they can easily penetrate the insect gut wall and bind to the sulfur and phosphorus group of deoxyribonucleic acid, causing cell death by interfering with normal functioning like replication. The mechanism of action of AgNPs towards larvae is poorly understood, with just a small number of publications available^5–9^. The effects of NPs on mosquito larvae were studied by Kumar et al. in terms of morphological, biochemical, physiological, and molecular alterations^10^.

Infectious illnesses, in general, constitute a severe danger to public health across the globe, particularly when antibiotic-resistant probiotic pathogens evolve. Gram-positive and Gram-negative strains of bacteria are both regarded to be a substantial public health concern. Antibiotics were used to manage diseases in both the community and hospital settings for many years^11–13^.

*Mollugo oppositifolia *L., often known as slim carpet weed, belongs to the *Molluginaceae* species (English). It's a thin, widespread, sleek, branching annual plant with 20 to 30 cm high branches that thrives in both dry and wet environments. Leaves are alternate or leathery, in tendrils of 4–5, uneven, oblanceolate or linear-lanceolate or occasionally rounded or sharp and apiculate at the apex, greatly tapering into the inconspicuous petiole. Flowers are white and borne in two or more axillary fascicles. Capsules are ellipsoid in shape and contain many dark brown seeds. Creaper and undesired roots^14^. In ethnomedicine, the herb is used for stomachic, earache, aperients, and skin problems. The leaves have a harsh flavor and are antiseptic. Mollugo subspecies have been shown to have antibacterial, anticancer, anti-inflammatory, and hepatoprotective properties^15^.

Due to their fascinating properties, nano-structure materials have drawn significant attention in recent years. Among these elements, much focus is drawn to research on fundamental characteristics and functional applications of transition-metal oxides^16–19^. Among the transition-metal oxides, cobalt oxides, Co_3_O_4_ and CoO are flexible materials that are stable in the natural environment^20,21^. In recent years, owing to their possible applications, much effort has been guided towards the synthesis and investigation of Co_3_O_4_ and CoO nanostructures^22–24^. Co_3_O_4_ is the thermodynamically stable type of cobalt oxide below 1164 K in ambient air, while Co_3_O_4_ is decomposed into CoO above this temperature^25^. Co_3_O_4_ is a natural spinel^26^ at room temperature and has several possible applications in gas sensors, magnetic materials, catalysts, and absorbers of solar energy^27–30^. Several processes, such as oxidation, microwave-assisted hydrothermal, ultrasonic, and hydrothermal have recently been developed for the preparation of Co_3_O_4_^31–34^. CoO, on the other hand, crystallises in the structure of rock salt and has possible uses in many areas, such as lithium battery anodes, pigments, magnetoresistant reading heads, and gas sensors^35–37^. While there are a few studies on the synthesis of CoO in bulk form, through simple methods, this compound is hard to acquire in pure form, mostly polluted with Co_3_O_4_ and Co metal.

Cobalt nanoparticles (Co NPs) have gained a lot of interest recently owing to their unique electrical and magnetic characteristics and lower cost compared to noble metal nanoparticles (NPs)^38,39^. Biomedical researchers have investigated the potential of CoNPs as therapeutic agents for the therapy of disorders like microbial infection^40,41^. At low concentrations, CoNPs are safe for the body, have potent antimicrobial and antifungal activities, and fewer adverse effects than antibiotics^42,43^.

Yin and Wang demonstrated that in the existence of surfactant Na(AOT) at 130 °C in air, decomposition of Co_2_(CO)_8_ in toluene occurs in CoO nanocrystals combined with Co_3_O_4_ and Co^44^. Ye et al. developed CoO nanomaterials under solvothermal conditions by an esterification reaction^45^. Ghosh et al. synthesised pure CoO nanoparticles under solvothermal conditions by the decomposition of Co(II) cupferronate in decalin at 270 °C^46^. Very recently, via a spry roasting process, Guo et al. prepared CoO particles using the CoCl_2_ solution^47^. A basic synthesis strategy, different from those described above, is suggested in this article. Through green chemistry approach *Mollugo oppositifolia *L. aqueous leaf extract assisted strategy, we will report on the synthesis process of Co_3_O_4_ nanoparticles and evaluation of its mosquito larvicidal and antimicrobial activities.

## Results and discussion

### Physiochemical characterization of biosynthesized Co_3_O_4_ NPs

**Figure 1:**
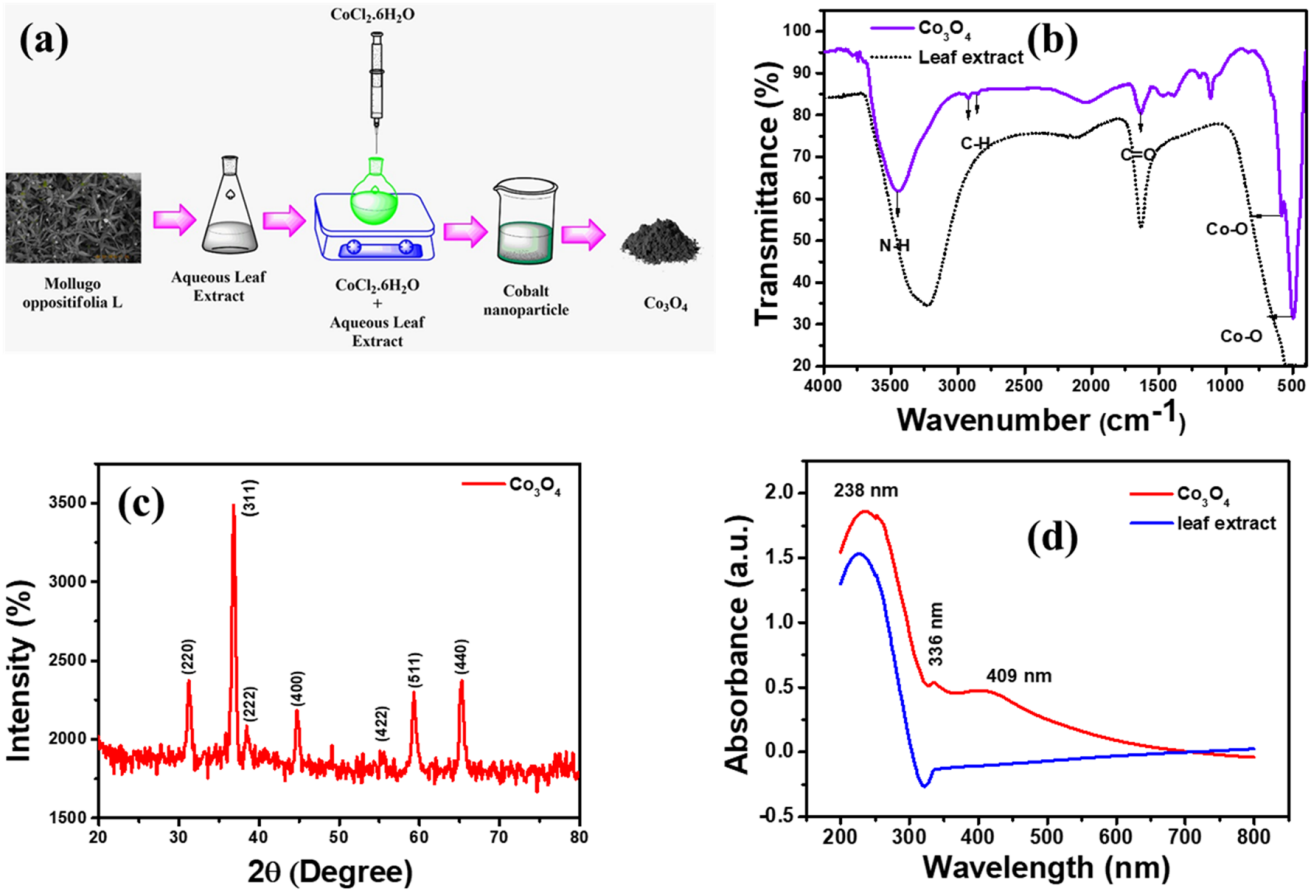
(**a**) The schematic representation of biosynthesized Co_3_O_4_ nanoparticles. (**b**) FT-IR spectra. (**c**) XRD spectra and (**d**) UV–visible spectra of biosynthesized Co_3_O_4_ nanoparticles.

The detailed schematic representation of biosynthesized Co_3_O_4_ nanoparticles is shown in Fig. 1a. FTIR spectra are often collected between 400 and 4000 cm^−1^. The FT-IR spectra of the biosynthesized Co_3_O_4_ NPs are shown in Fig. 1b. A wide peak at 3465.93 cm^−1^ indicates the presence of the N–H group, which may have appeared as an amine moiety. The presence of C–H functional group alkanes is indicated by a band between 2800 and 3000 cm^−1^. C=O was identified at 1644.01 cm^−1^ from the PVP moiety, as shown by spectral peaks. Both tetrahedral and octahedral Co–O vibrations are confirmed by the bands at 509.59 cm^−1^ and 584.80 cm^−1^, respectively. The functional groups of the capping agent and the synthesis of Co_3_O_4_ nanoparticles were authenticated by FT-IR analysis. In addition, The XRD pattern was employed to analyze the phase purity and crystalline nature of biosynthesized Co_3_O_4_ NPs, as shown in Fig. 1c.

Pure face-centered cubic spinel phase structure of Co_3_O_4_ NPs was identified by diffraction peaks at 2 = 31.2°, 37.6°, 38.7°, 44.8°, 55.6°, 59.8°, and 66.3°, which were indexed to (220), (311), 222), 400, (422), 511, and 440) planes. Standard Co_3_O_4_ NPs were found to have diffraction peaks that were quite similar to those produced. The diffraction peaks all rather closely match the typical distribution for pure Co_3_O_4_ nanoparticles (JCPDS No. 00-042-1467). Certain peaks indicative of impurities have been detected. These pronounced peaks show that the resulting nanoparticles are very crystalline. The average crystallographic size may be determined from the observed primary diffracted peak by using the Scherer equation,

$${\mathrm{D}}_{(\mathrm{hkl})}=\frac{{\text{k}\uplambda }}{{\upbeta \cos \uptheta }}$$

where, D_(hkl)_ is the typical crystallographic dimension, k is shape constant (0.89), λ is the wavelength of the incident x-ray (Cukα source, λ = 0.15405 nm), β is the full width half maximum (FWHM), θ is the incident angle of x-ray. A 22.70 nm Co_3_O_4_ crystal was successfully produced.

Furthermore, UV–visible spectroscopy was used to investigate the optical absorption characteristics of the biosynthesized Co_3_O_4_ NPs at room temperature, and the results are shown in Fig. 1d. The formation of Co_3_O_4_ NPs is indicated by a UV–visible absorption peak at 409 nm. Our research focuses on two distinct absorption bands, between 200 and 340 nm and 336 and 409 nm. According to published research, these bands can be assigned to the O_2_^–^ → Co_2_^+^ and O_2_^–^ → Co_3_^+^ charge transfer processes, respectively. In addition, the plant extract shows an absorption peak at around 238 nm which confirms the successful formation of biosynthesized Co_3_O_4_ nanoparticles.

### Morphological and elemental analysis of biosynthesized Co_3_O_4_ Nps

**Figure 2:**
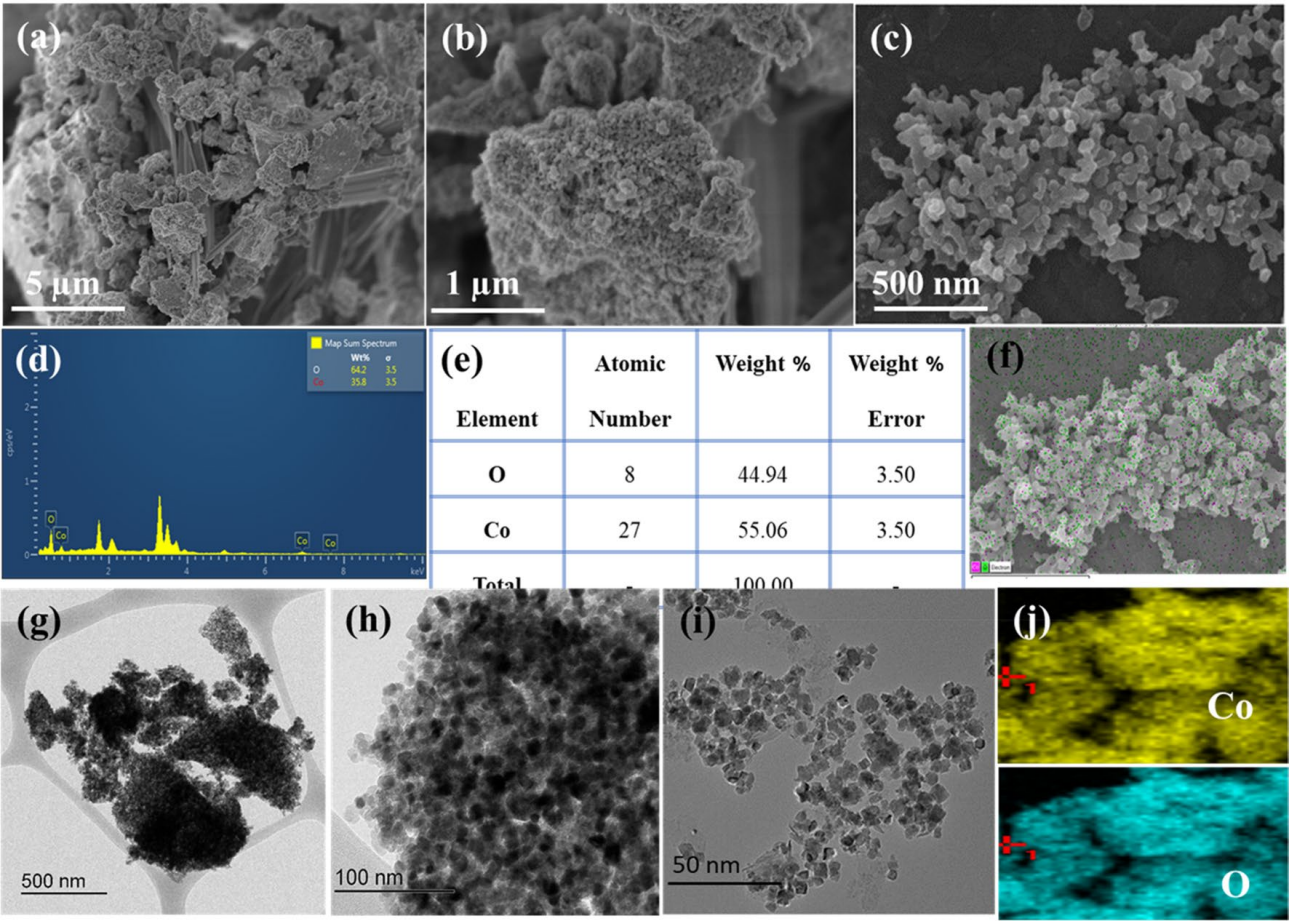
(**a**–**c**) FE-SEM morphology images (**d**–**f**) EDX spectra and elemental mapping (**g**–**j**) HR-TEM images and (**j**) elemental mapping of biosynthesized Co_3_O_4_ nanoparticles.

Scanning electron microscopy was used to regulate the resulting Co_3_O_4_ NPs' size and form (SEM). Scanning electron microscopy (SEM) scans confirmed the spherical shape of the biosynthesized Co_3_O_4_ NPs (Fig. 2a–c). The biosynthesized Co_3_O_4_ NPs were distributed in the wild as a population of uniformly sized particles. In addition, the EDX analysis confirmed the atomic composition of the biosynthesized Co_3_O_4_ NPs. The presence of cobalt and oxygen peaks in the EDX spectra confirmed that the material was really Co_3_O_4_ NPs (Fig. 2d). There was 3.58% cobalt and 64.20% oxygen by molecular weight. Extra peaks in the EDX spectra might be due to bioorganic or contaminant presence in the solution. The chemical composition of the biosynthesized Co_3_O_4_ nanoparticles is shown in Fig. 2e. Studies using scanning electron microscopy to map the nanoparticle proved its identity as Co_3_O_4_ (Fig. 2f). Cobalt is represented by the pink dots, whereas Oxygen is shown by the green ones. Detailed morphological features and chemical compositions of biosynthesized Co_3_O_4_ nanoparticles were analyzed by HR-TEM, obtained results are shown in Fig. 2g–j. TEM images demonstrate the existence of aggregated polycrystalline particles with restricted size distribution and a spherical shape. The particle size from TEM images is well-matched with the particle size predicted by the Debye–Scherrer equation. Figure 2j shows the elemental mapping of biosynthesized Co_3_O_4_ nanoparticles, which confirms the presence of Co and O elements with uniform distribution.

### Larvicidal activity

The biosynthesized Co_3_O_4_ particle (**2**) was much more active in contrast to *Culex quinquefasciatus* with an LD_50_ value of 34.96 μg/mL than the aqueous plant extract (**1**) and control Permethrin, which had LD_50_ values of 82.41 and 72.44 μg/mL, respectively. The aqueous plant extract (**1**) showed the least amount of activity against *Culex quinquefasciatus*, with LD_50_ values that were respectively 82.41 μg/mL. This was one of the samples that was tested. When compared to the positive control Permethrin, which had an LD_50_ value of 72.44 μg/mL, the manufactured Co_3_O_4_ nanoparticle (**2**) exhibited very high levels of activity, while the aqueous plant extract (**1**) exhibited only moderate levels of activity. The findings are shown in Table 1 below.

**Table 1:** Larvicidal activity of aqueous plant extract (**1**) and synthesized Co_3_O_4_ nanoparticle (**2**).

Comp. no.	Mortality (%)/concentration (μg/mL)^a^				LD_50_ (μg /mL)
	100	75	50	25	
**1**	60.20 ± 1.64	46.78 ± 0.24	24.16 ± 1.42	10.86 ± 0.84	82.41
**2**	100 ± 0.00	82.14 ± 1.25	68.48 ± 0.68	32.86 ± 1.20	34.96
**Permethrin**	70 ± 0.67	46 ± 1.29	30 ± 1.78	16 ± 0.98	72.44
**DMSO**	0.0 ± 0.0	0.0 ± 0.0	0.0 ± 0.0	0.0 ± 0.0	0.0 ± 0.0

^a^Values were the averages of three trials ± SD.

### In vitro antibacterial activity

**Figure 3:**
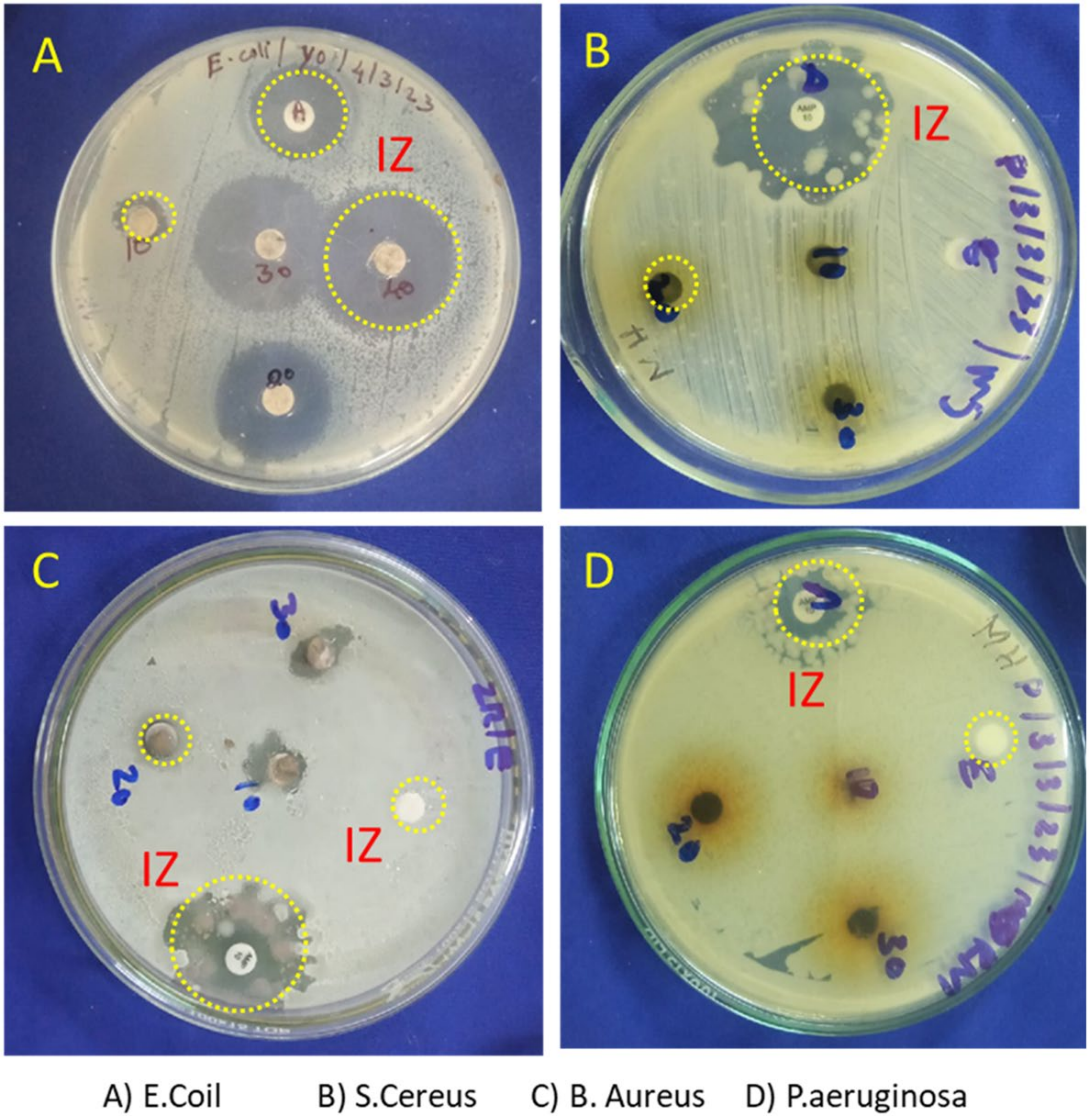
Antibacterial image plates of biosynthesized Co_3_O_4_ nanoparticles (IZ—inhibition zone).

The antibacterial activity of ciprofloxacin was tested in vitro against four different bacteria: two Gram-negative (*E. coli* and *Pseudomonas aeruginosa*) and two Gram-positive (*S. aureus* and *Bacillus cereus*). The MIC values were determined using the standard agar method. Figure 3 and Table 2 displays the MIC values for both the synthesized Co_3_O_4_ nanoparticle (**2**) and the aqueous leaf extract of *Mollugo oppositifolia L.* (**1**). Compared to an aqueous leaf extract of *Mollugo oppositifolia *L., the antibacterial activity of the synthetic Co_3_O_4_ nanoparticle is much greater (**1**). Co_3_O_4_ nanoparticle (**2**) has significant antibacterial activity against *E. coli*, with a minimal inhibitory concentration (MIC) of 23.60 μg/mL compared to a MIC of 25.00 μg/mL for the control ciprofloxacin. Co_3_O_4_ nanoparticle (**2**) had a substantially lower MIC of 26.56 μg/mL than the 50.00 μg/mL of the control ciprofloxacin against *B. cereus*. The MIC for Co_3_O_4_ nanoparticle (**2**) against *P. aeruginosa* and *S. aureus* was 34 and 28 μg/mL, respectively, which is somewhat higher than the MIC for normal ciprofloxacin. Synthetic Co_3_O_4_ nanoparticle (**2**) substantially surpassed the reference activity in killing *E. coli* and *B. cereus* when compared to the gold standard antibacterial Ciprofloxacin.

**Table 2:** In vitro antibacterial activity of a synthetic aqueous plant extract (**1**) and Co_3_O_4_ nanoparticle (**2**).

Comp. no.	MIC^a^ µg/mL			
	*E****. ****coli*	*P****. ****aeruginosa*	*S****. ****aureus*	*B****. ****cereus*
**1**	32.10 ± 0.24	40 ± 1.23	32 ± 0.62	52.23 ± 1.24
**2**	23.60 ± 1.25	34 ± 0.21	28 ± 1.12	26.56 ± 0.56
**Ciprofloxacin**	25.00 ± 0.95	30 ± 0.0	20 ± 0.0	50.00 ± 1.75

^a^Values were the averages of three trials ± SD.

### Antifungal activity

**Figure 4:**
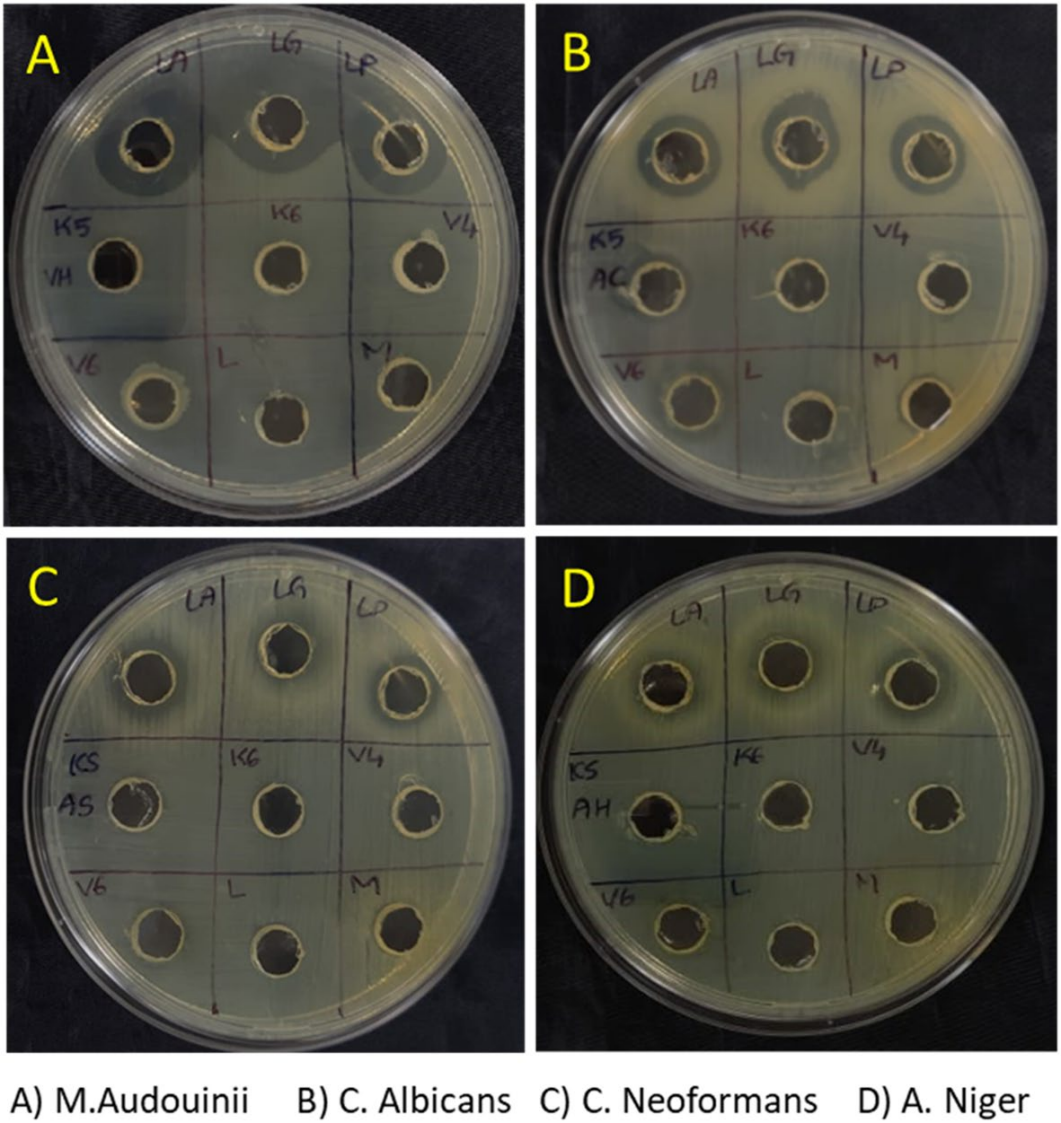
Antifungal image plates of biosynthesized Co_3_O_4_ nanoparticles.

Fresh leaf extract **1** of *Mollugo oppositifolia *L. and synthetic nanoparticle **2** were both examined for their ability to inhibit the activity of four different types of fungi. When compared to compound **1**, compound **2** is substantially more effective against fungi. Compound **2** effectively combats the fungal infections caused by *Candida albicans* and *Malassezia audouinii*. The minimal inhibitory concentration (MIC) of Compound **2** for *C. albicans* growth was 01 μg/mL, which was much lower than the control clotrimale (02 μg/mL). Compound **2** has an even lower MIC against *M. audouinii* than clotrimale, which has a MIC of 4 μg/mL. You can see the results in Table 3 and Fig. 4.

**Table 3:** Antifungal activity of synthetic aqueous plant extract (**1**) and Co_3_O_4_ nanoparticle (**2**).

Compds	MIC^a^ (μg/mL)			
	*Aspergillus niger*	*Candida albicans*	*Microsporum audouinii*	*Cryptococcus neoformans*
**1**	52	18	32	24
**2**	46	01	02	18
**Clotrimale**	01	02	04	05

^a^Value were the means of three replicates ± SD.

### Docking studies

**Figure 5:**
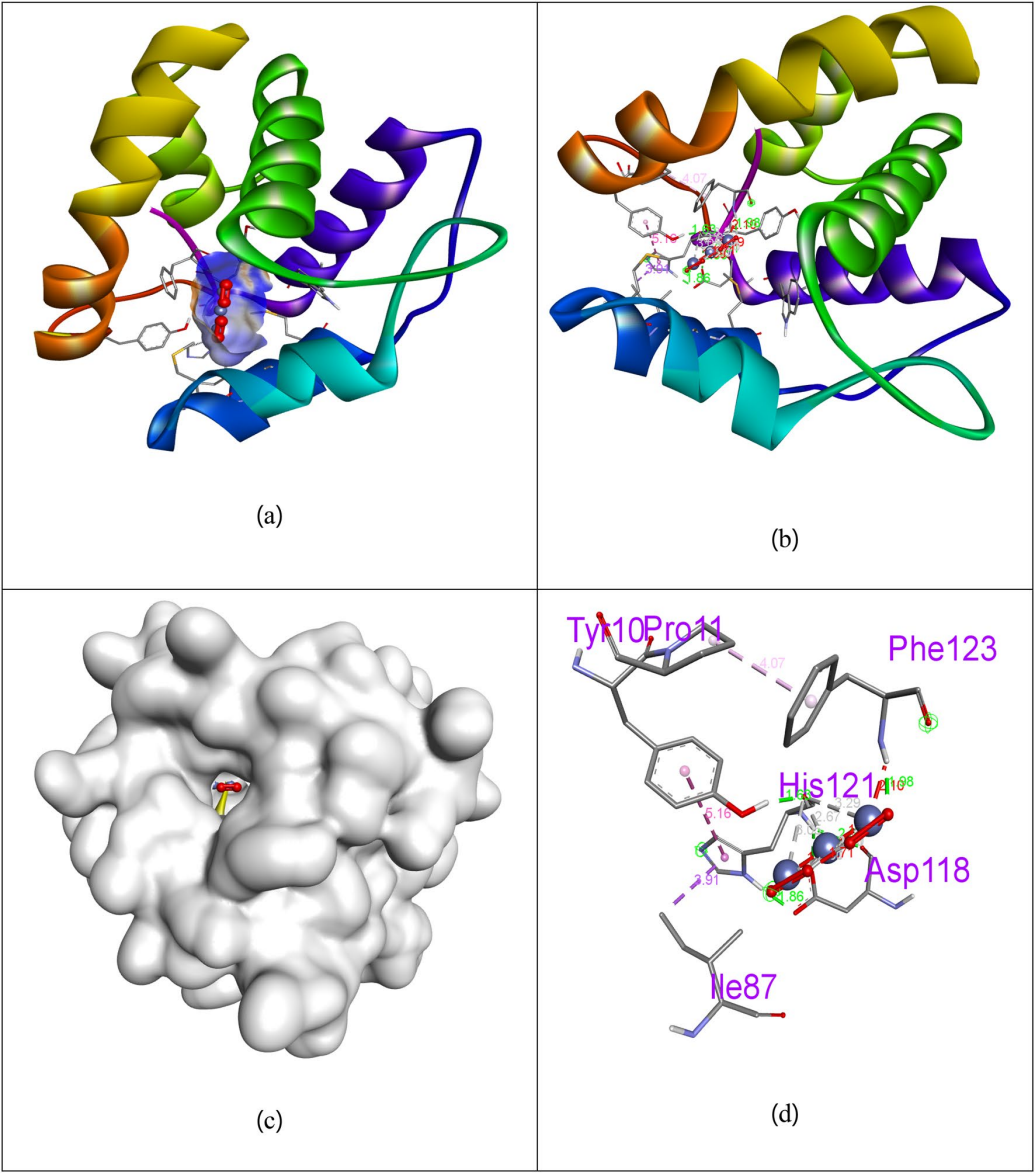
Docked complex (**a**), Helix (**b**), molecular surface (**c**), and 3D (**d**) interaction modes of compound **Co**_**3**_**O**_**4**_ Nps within the binding site of 3OGN protein.

**Figure 6:**
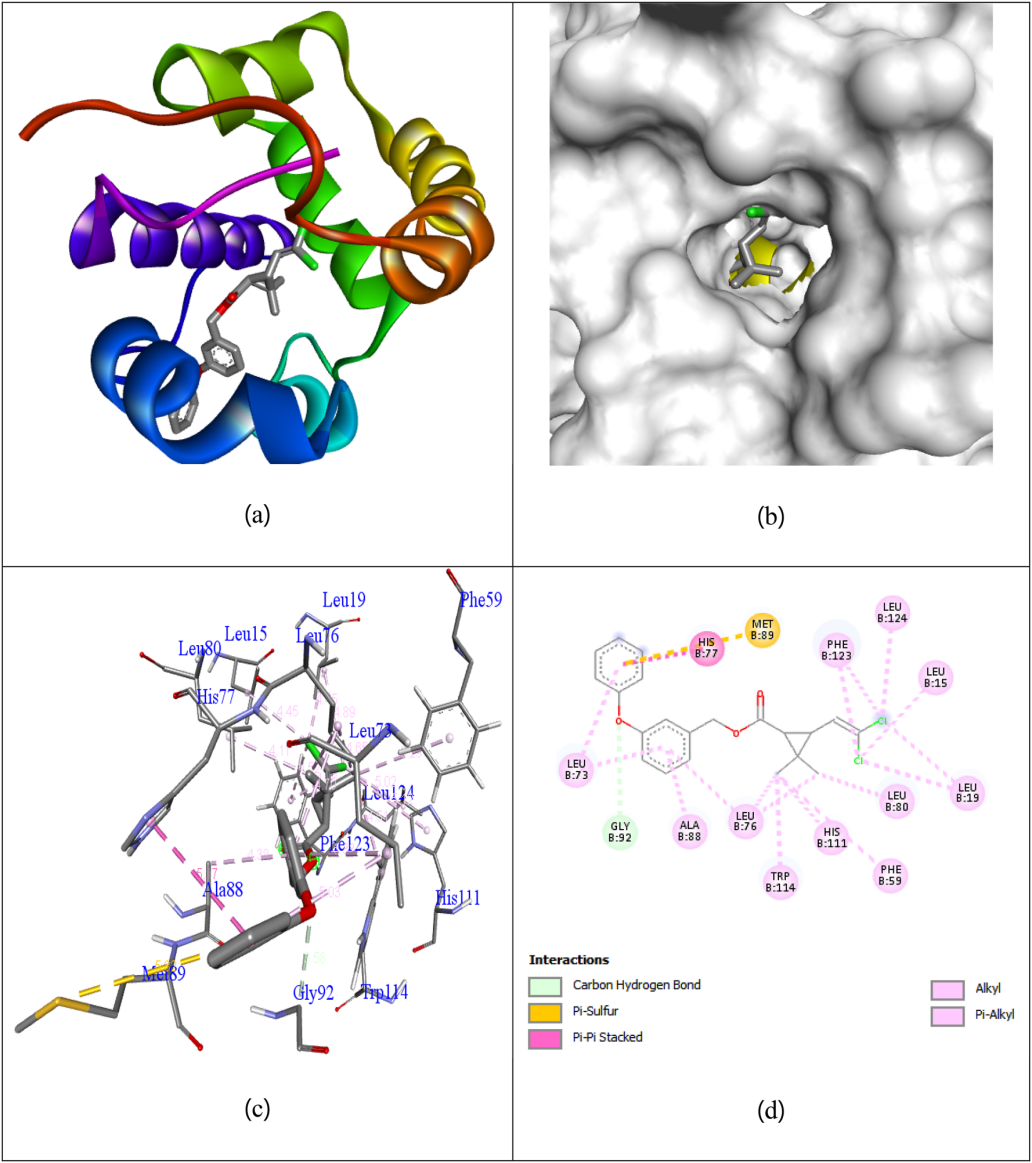
Docked complex (**a**), molecular surface (**b**), 3D (**c**), and 2D (**d**) interaction modes of control **permethrin** within the binding site of 3OGN protein.

The **Co**_**3**_**O**_**4**_ Nps, were studied for their docking behavior with 3OGN protein via Autodock Vina program. The **Co**_**3**_**O**_**4**_ Nps, shows excellent binding affinity (− 8.5 kcal/mol) than **permethrin** with the binding affinity of (− 4.4 kcal/mol) in 3OGN protein respectively. Hydrogen bonding is one of the significant factor in the stability of protein–ligand bonding, and the favorable bond distance amongst the H-donor and the H-acceptor atoms is less than 3.5 Å. The hydrogen bond distances of **Co**_**3**_**O**_**4**_ Nps, were less than 3.5 Å in respective 3OGN protein signifies strong hydrogen bonding. **Co**_**3**_**O**_**4**_ Nps, forms three hydrogen bond interaction with the receptor 3OGN. The amino acid residue Asp118 (bond length: 2.10), His121 (bond length: 1.63) and Phe123 (bond length: 1.98) were involved in hydrogen bonding contacts. The amino acid residues Tyr10, Pro11, and Ile87 were involved in hydrophobic interactions. The interactions of **Co**_**3**_**O**_**4**_ Nps with 3OGN protein were shown in Fig. 5**.** The control **permethrin** did not form any Hydrogen bond interaction with the receptor 3OGN. The amino acid residues Leu15, Leu19, Phe59, Leu76, Leu76, His77, Leu80, Ala88, Met89, Gly92, His111, Trp114, Phe123 and Leu124 were involved in hydrophobic interactions. The interactions of compound **permethrin** with 3OGN protein were shown in Fig. 6**.** The results shows that **Co**_**3**_**O**_**4**_ Nps having remarkable inhibition ability than control **permethrin** in larvicidal mosquito odorant binding protein 3OGN. The results were summarized in Table 4.

**Table 4:** Molecular docking interaction of **Co**_**3**_**O**_**4**_ Nps against mosquito odorant binding protein 3OGN.

Compounds	Mosquito odorant binding protein 3OGN		
	Binding affinity (kcal/mol)	No. of H-bonds	H-bonding residues
**Co**_**3**_**O**_**4**_ Nps	− 8.5	3	Asp118, His121, Phe123
**Permethrin**	− 4.4	0	–

## Conclusions

Progressive experience is an imperative of the hour for successful mosquito vector regulator. The current research highlights the recorded method of *Mollugo oppositifolia* L. aqueous leaf extract assisted synthesis of nanoparticles of Co_3_O_4_. Biosynthesized Co_3_O_4_ Nps were confirmed via UV–vis spectroscopy, Fourier-transform infrared spectroscopy, X-ray diffraction, scanning electron microscope, HR-TEM and mapping studies. In addition, Co_3_O_4_ nanoparticle was further evaluated for mosquito larvicidal, antibacterial and antifungal activities. When compared to an aqueous plant extract (**1**) and control Permethrin (LD50 = 82.41 and 72.44 μg/mL, respectively), the activity of the synthesized Co_3_O_4_ particle (**2**) against *Culex quinquefasciatus* was much higher. The synthesized Co_3_O_4_ nanoparticle **(2**) exhibits an antibacterial activity that is much greater than that of the control Ciprofloxacin in both the pathogens *E. coli* and *B. cereus*. When compared to the control Clotrimale, which had a MIC value of 2 μg/mL, the Co_3_O_4_ nanoparticles 2 had a MIC value of 1 μg/mL, which made them substantially more effective against *C. albicans*. In comparison, the antifungal activity of Co_3_O_4_ nanoparticles 2 against *M. audouinii* is much greater than that of Clotrimale, which has a minimal inhibitory concentration (MIC) value of 4 μg/mL. Consequently, Co_3_O_4_ nanoparticles might be a probable basis for emerging environmentally friendly bioactive compound, as well as ecological biopharmaceuticals and insecticides.

## Experimental

### Chemicals and bacterial strains

All chemical substances and solvents were purchased from Nice and Loba chemicals. High-purity solvents were utilized for synthesis processes without further purification. The bacterial strains *E. coli*, *Pseudomonas aeruginosa*, *S. aureus* and *Bacillus cereus* as well as different types of fungi were purchased from Iranian biological resource center, Pasture Institute of Iran.

### Resources of plant leaves

The *Mollugo oppositifolia* L. used in this study was gathered from several locations in the greater Chennai region. Leaf waste from recent harvests was used in the production of Co_3_O_4_ Np. The collection of plant material and related studies complies with relevant institutional, national, and international guidelines and legislation.

### Preparation of plant extract

The *Mollugo oppositifolia* L. plant species were used to generate the extract solution from the leaves of the plant. Greens from a plant that have been collected quite recently washed with deionized water and chopped very coarsely. Following the boiling of the plant material in 100 mL of distilled water at 100 °C, it was filtered and then stored at 4 °C for further examination.

### Biosynthesis of cobalt oxide nanoparticles

Preparing cobalt oxide nanoparticles began with dissolving CoCl_2_⋅6H_2_O (0.1 g) in a sufficient amount of deionized water, followed by the addition of 10 mL of a solution containing an extract of the *Mollugo oppositifolia* L. plant. Then, for 3 h at room temperature, the mixture was agitated at a speed of 1000 rpm using a magnetic stirrer. The pH of the reaction mixture was adjusted by adding a 1 mL solution of 10% NaOH to the mixture. The precipitate was filtered and then evaporated for 12 h, the oven was set to 150 °C and let to do its thing. Following collection, the powder was calcined for 3 h at 500 °C before being ground into a fine powder.

### Characterization studies

Cobalt oxide nanoparticles were synthesized by using ultrasonic assisted chemical precipitation technique. UV–Visible spectra was recorded with V-730 UV–visible Spectrophotometer at a wavelength range between 200 and 800 nm. FT-IR spectra was recorded with Fourier transform infrared spectrometer (FT/IR-6600) (CHI 1000C) in the range 4000–400 cm^−1^, Powder XRD was assessed with X'Pert Pro by PANalytical, FE-SEM with EDX and Mapping were done with FESEM sigma essential by Zeiss Microscopy. HR-TEM images and mapping images of the biosynthesized Co_3_O_4_ nanoparticle are obtained from HR-TEM (Hitachi). Molecular docking studies were used to inspect the interaction, binding mode between compounds Co3O4 Nps, Permethrin and the mosquito odorant binding protein using Autodock vina 1.1.2^28^. The crystal structure of mosquito odorant binding protein (PDB ID: 3OGN) was taken from Protein Data Bank (http://www.rcsb.org). The 3D assembly of the compounds Co3O4 Nps, and permethrin were achieved via ChemDraw Ultra 12.0 and Chem3D Pro 12.0 software. The input files for Autodock Vina were created by using Autodock Tools 1.5.6 program package. The search grid of 3OGN protein was fixed at center_x: 18.681, center_y: 49.66, and center_z: 11.409 with dimensions size_x: 22, size_y: 20, and size_z: 22 with spacing of 1.0 Å. The exhaustiveness value was set to 8. The other parameters were set to default for Vina docking and not mentioned. The compound having least binding affinity value is the best-scoring compound and the results were visually analyzed using Discovery studio 2019 program.

### Larvicidal activity

The biosynthesized Co_3_O_4_ nanoparticle was further assessed for larvicidal activity against south urban mosquito larvae Culex quinquefasciatus. Appraisals were made on 'a dead/alive premise. The evaluations are based on a rate size that ranges from 0 to 100, where 0 represents no activity at all and 100 represents outright murder. The bioassay was repeated a number of times, and the results of the bioactivity test served as the standard for each of these replications. The characteristics are compared with those of the positive control substance permethrin. The LD50 values of a few different dynamic title mixtures were determined by probit analysis, and the results were analyzed through the use of the SPSS v16 software.

### Larvicidal activity against mosquito (*Culex quinquefasciatus*)

The aqueous plant extract (**1**) and combined Co_3_O_4_ nanoparticle (**2**) were assessed for larvicidal activity against south urban mosquito larvae Culex quinquefasciatus. The assessment of larvicidal activity at the starter test convergence of 100 μg/mL in contrast to the 4th instar south-urban mosquito larvae Culex quinquefasciatus through water immersion strategy beneath relative stickiness 50–70%, photoperiod of 10:14 (light: dark), and temperature of (27 ± 2) °C. The tests were set up at the convergences of 100, 75, 50, 25 μg/mL by utilizing dissolvable Dimethylsulfoxide (DMSO). All the test measuring glasses comprising twenty Culex quinquefasciatus were assessed for 24 h later handling. The outcomes were documented by average percent mortality.

### Antibacterial activity

Kirby Bauer tested the antibacterial effectiveness of an aqueous plant extract (**1**) and a mixed Co_3_O_4_ nanoparticle suspension against *Staphylococcus aureus*, *Escherichia coli*, *Klebsiella pneumophila*, and *Pseudomonas aeruginosa* in vitro (**2**). Discs are the preferred method for dispersing molecules^48^. The antibacterial activity of ciprofloxacin was utilized as a standard. Bacteria were cultured on petri plates using nutrient agar. All of the synthesis was carried out in DMSO, and the chemicals were held on a filter paper disc that measured 5 mm in diameter and 1 mm in thickness. After 24 h incubation at 37 °C, the discs were tested for antibacterial activity by measuring the size of the inhibitory zone^49,50^ surrounding each one put on the plates that had previously been implanted. Minimum inhibitory concentrations (MIC) were used to compare the antibacterial activity of an aqueous plant extract (**1**) and Co_3_O_4_ nanoparticles (**2**).

### Antifungal activity

The standardized disc–agar diffusion technique^51,52^ was used to assess the antifungal activity of aqueous plant extract (**1**) and combined Co_3_O_4_ nanoparticle (**2**). Microsporum audouinii (MTCC-8197), Candia albicans (MTCC-227), Cryptococcus neoformans (recultured) and Aspergillus niger (MTCC-872) were used to test antifungal activity. The materials were sterilised by filtering using 0.22 m Millipore filters after being dissolved in 10% dimethyl sulfoxide (DMSO) to a desired concentration of 30 mg/mL. Antifungal studies were then performed utilising disc diffusion technique with 100 L of solution containing 104 spore/mL of fungi dispersed over PDA medium. The discs (6 mm in diameter) were treated with 10 mL of the samples (300 g/disc) and put on the infected agar. The typical medicine was **clotrimale**. 10 percent DMSO was used to make negative controls. For fungus specimens, the inoculation plates were then incubated at 37 °C for 72 h. Fungi linked with plants were cultured at 27 °C. The zone of inhibition against the tested strains was used to assess antifungal activity. In this study, each test was carried out twice.

## Data Availability

The datasets used and/or analysed during the current study available from the corresponding author on reasonable request.
